# Potential for perceived failure of stratospheric aerosol injection deployment

**DOI:** 10.1073/pnas.2210036119

**Published:** 2022-09-27

**Authors:** Patrick W. Keys, Elizabeth A. Barnes, Noah S. Diffenbaugh, James W. Hurrell, Curtis M. Bell

**Affiliations:** ^a^Department of Atmospheric Science, Colorado State University, Fort Collins, CO 80523;; ^b^School of Global Environmental Sustainability, Colorado State University, Fort Collins, CO 80523;; ^c^Doerr School of Sustainability, Stanford University Stanford, CA 94305;; ^d^International Programs Department, United States Naval War College, Newport, RI 02841

**Keywords:** climate change, internal climate variability, solar geoengineering, perception, mitigation

## Abstract

As anthropogenic activities warm the Earth, the fundamental solution of reducing greenhouse gas emissions remains elusive. Given this mitigation gap, global warming may lead to intolerable climate changes as adaptive capacity is exceeded. Thus, there is emerging interest in solar radiation modification, which is the process of deliberately increasing Earth’s albedo to cool the planet. Stratospheric aerosol injection (SAI)—the theoretical deployment of particles in the stratosphere to enhance reflection of incoming solar radiation—is one strategy to slow, pause, or reverse global warming. If SAI is ever pursued, it will likely be for a specific aim, such as affording time to implement mitigation strategies, lessening extremes, or reducing the odds of reaching a biogeophysical tipping point. Using an ensemble climate model experiment that simulates the deployment of SAI in the context of an intermediate greenhouse gas trajectory, we quantified the probability that internal climate variability masks the effectiveness of SAI deployment on regional temperatures. We found that while global temperature was stabilized, substantial land areas continued to experience warming. For example, in the SAI scenario we explored, up to 55% of the global population experienced rising temperatures over the decade following SAI deployment and large areas exhibited high probability of extremely hot years. These conditions could cause SAI to be perceived as a failure. Countries with the largest economies experienced some of the largest probabilities of this perceived failure. The potential for perceived failure could therefore have major implications for policy decisions in the years immediately following SAI deployment.

## Significance

Even if aggressive mitigation policies are implemented soon, climate change impacts will worsen in the coming decades. One proposed response is stratospheric aerosol injection (SAI), which would reflect a small amount of the sun’s energy back to space, thereby cooling the planet. This approach is broadly considered to be relatively inexpensive and straightforward to deploy, and global cooling could occur rapidly. However, on regional scales, internal climate variability is likely to dominate over SAI forcing. This means that in the decade after SAI is deployed, many regions of the world could locally experience even higher temperatures. Our study provides conceptual insight for the possible perception of the failure of SAI or other climate mitigation strategies.

Anthropogenic climate change, primarily driven by increasing concentrations of atmospheric greenhouse gasses, has caused Earth’s global mean temperature to reach its warmest level in at least the last 2,000 y (1). This global warming may exceed 1.5 °C above preindustrial temperatures later this decade, at least for a short period of time, and most years are likely to exceed the 1.5 °C threshold by 2040 across a range of emissions scenarios (1). By the middle of this century (2041–2060), warming in excess of 2.0 °C would be reached under intermediate, high, and very high emission scenarios (1), and current policies have the world on track to warm by roughly 3.0 °C by the end of the century (2). Moreover, emissions scenarios that target global temperature stabilization at either 1.5 or 2.0 °C require net-zero carbon emissions trajectories, which in practice will necessitate new and enormously scaled-up carbon dioxide removal technology (3).

In parallel with global policy shortfalls, current levels of warming are driving substantial impacts on human and natural systems (4). For example, climate change is already leading to intensification of extreme events such as extreme heat, heavy rainfall, intense droughts, extreme wildfire weather, and marine heat waves (4). These and other climate changes are leading to a broad suite of impacts, such as migration of ecological niches (5), increases in global tree mortality (6), increases in financial losses from extremes (e.g., 7), and amplification of existing economic inequality (8) and social injustices (9). Furthermore, there is the possibility that biogeophysical tipping points may lead to new states in key Earth systems, such as irreversible Antarctic ice loss, tropical rainforest dieback, and slowing ocean circulations (10). These so-called tipping points are highly uncertain—in terms of whether, when, and how they may occur (1). Despite this uncertainty, there is paleoclimate evidence that tipping points have been crossed in the past, and emerging evidence suggests that they could be crossed as a result of anthropogenic change (11–13).

To possibly grant humanity additional time to sufficiently reduce greenhouse gas emissions, lessen the existing negative impacts of climate change, and avoid transgression of irreversible tipping points, there is renewed interest in developing an international research agenda on solar radiation modification (SRM)—a speculative form of climate change response that has the potential to offset human-induced warming by reflecting a small amount of solar energy back to space before it enters and warms the planetary environment (14).

There are numerous challenges for advancing SRM science and research. First, there are substantial ethical questions concerned with committing future generations to an uncertain technology and the potential burden of continuing climate intervention well into the future (15) or deciding when and how to ramp down SRM deployment (16–19). Second, there are important concerns related to how climate intervention may drive changes in essential Earth system processes (20, 21). Third, there are concerns that the negative consequences arising from SRM would disproportionately burden populations that are systematically already burdened by climate change impacts, global dispossession of resources, and wealth inequality (22, 23). Research investigating public opinion has found considerable heterogeneity in attitudes toward either research or use of climate intervention (24).

In addition to these social challenges, there exist basic scientific questions about how to distinguish the climate effects of SRM from anomalies driven by internal variability of the Earth system (25, 26). This variability can lead to substantial short-term variation in socially relevant climate phenomena, such as the frequency of extreme hot and cold spells (27), the severity of drought (28), the path of the midlatitude storm tracks (29), changes in regional temperature and precipitation (30), the state of Arctic Sea ice (31), or the strength of tropical modes of variability such as the El Niño Southern Oscillation (32) or the Madden-Julian Oscillation (33). Research on the interaction between human-induced climate impacts, or “signals,” and internal climate variability, or “noise,” is a critical area of climate change science, not least for supporting policymakers and the public in navigating the expectations of climate change action against a backdrop of an internally varying climate system (34).

Stratospheric aerosol injection (SAI) is the SRM strategy of releasing particles into the stratosphere to slow, pause, or reverse global warming (35). While climate simulations provide evidence that the long-term result of SAI could lead to stabilized global temperatures (17), the impacts of SAI may be regionally heterogeneous, with temperature and precipitation varying considerably (36–39). Moreover, internal climatic variability may mask the short-term perceived effectiveness of SAI; that is, it is possible that while SAI could successfully stabilize mean global temperatures, the perceived effectiveness on *regional* scales may be overwhelmed by local climatic variability over the short term. Psychologically, a climate change–related event connects to people’s perceptions most clearly when it is directly and locally relevant (40, 41). Moreover, people who are residents of a specific location may tacitly incorporate 10-y trends in their perception of changes in climate (42). Hence, local changes in climate—such as continued warming or the occurrence of extreme events—may cause climate interventions such as SAI to be perceived as a failure. Given the potential for SAI to abruptly cease and the likelihood of rapid climate change following such cessation (e.g., 19, 43), the perception of failure carries particular risks.

If SRM is ever pursued, it will likely be for a specific social or geophysical aim (22). This may include halting an anticipated geophysical tipping point [such as accelerated Antarctic ice loss (44), permafrost melting, or forest die-off] or lessening the impacts of extremes such as deadly heat waves in large population centers (45). Yet, if climate variability were to mask the short-term perceived effectiveness of climate intervention, it could undermine coordinated, international policy action to address climate change broadly (46). Understanding the masking effects of climate variability on regional scales will thus be critical for interpreting the potential perceived success of any SRM strategy in the immediate years following deployment.

To systematically distinguish the different possible outcomes associated with the masking effect of internal climate variability, we introduce a set of archetypal regional responses that could unfold under SAI. These archetypes are motivated by the fact that in the period prior to SAI deployment, a given region could be warming or not due to internal climate variability, even in the context of global-scale warming (47). Similarly, following deployment, that region could either experience warming or not, even if the global temperature is stabilized. Thus, we defined four archetypes of perceived success of climate intervention based on four categories of pre- and postdeployment experience: 1) Rebound Warming (i.e., no warming followed by warming); 2) Continued Warming (i.e., warming followed by more warming); 3) Stabilization (i.e., no warming either before or after deployment); and 4) Recovery (i.e., warming followed by no warming). The phenomena Rebound Warming and Continued Warming could both be locally perceived as a failure of SAI to deliver on its intended purpose; hence, throughout the rest of this work, the phrase “perceived failure” refers to the combination of these two archetypes.

**Fig. 1.**
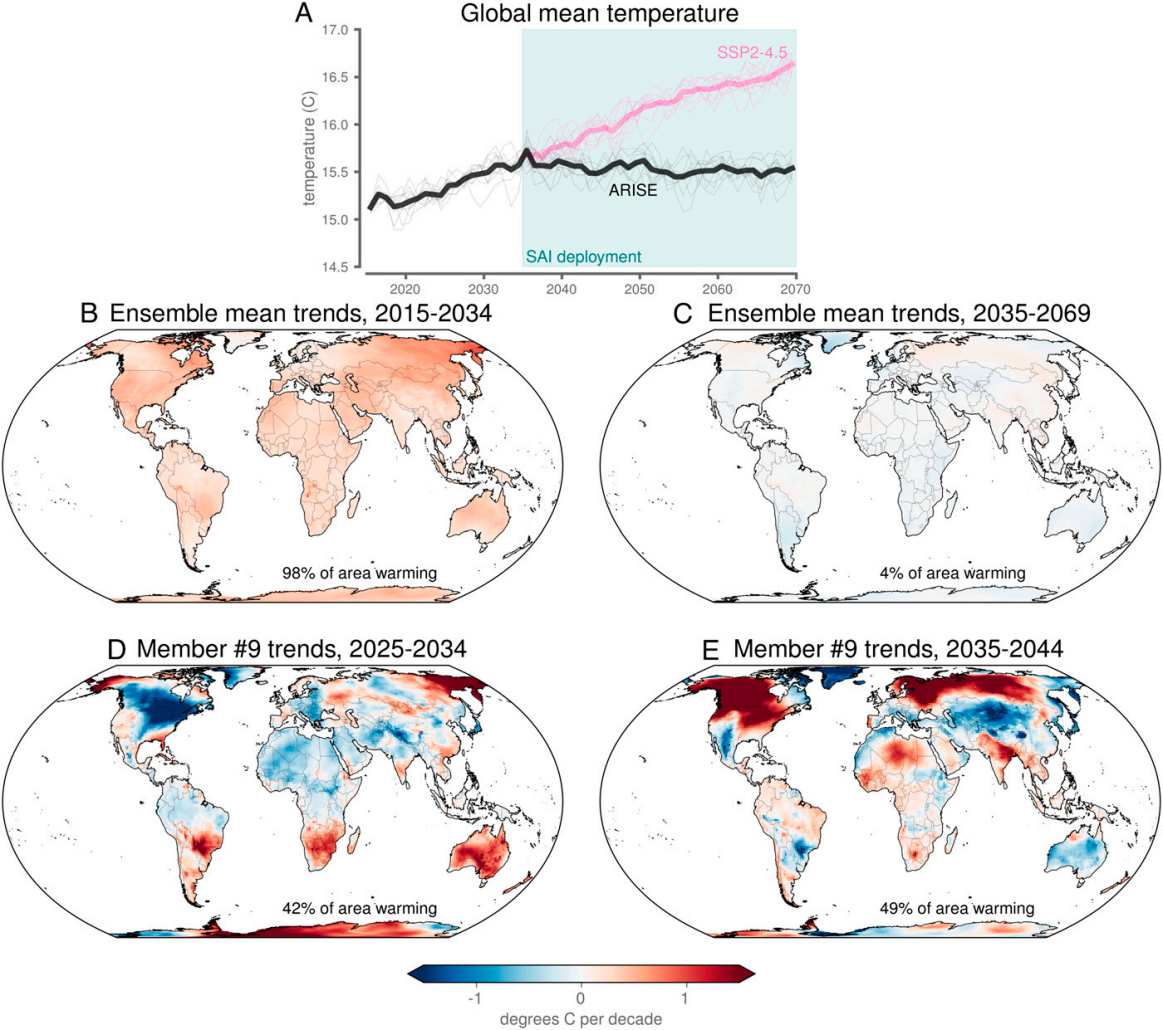
Surface temperature trends. (*A*) Global mean surface temperature. Gray lines denote individual ensemble members, and the black line denotes the ensemble mean. (*B* and *C*) Ensemble-mean trends over years 2015 to 2034 under SSP2-4.5 (*B*) and 2035 to 2069 (*C*) with ARISE-SAI deployment. (*D* and *E*) Trends over the predeployment decade (*D*) and postdeployment decade (*E*) for ensemble member #9. (*B–D*) The percentage in the bottom of the maps denotes the percentage of land area that exhibited warming trends as defined in the text.

Past research into global SRM strategies employed climate or Earth system models to simulate how the natural system might respond to different intervention approaches (48). Here, we leveraged just one of them: the Assessing Responses and Impacts of SRM on the Earth system with Stratospheric Aerosol Injection (ARISE-SAI) ensemble carried out with the Community Earth System Model, version 2 (CESM2) (49). ARISE-SAI simulates a plausible deployment of SAI, designed to hold global mean temperature at 1.5 °C above preindustrial conditions in the context of the Shared Socioeconomic Pathway 2 (SSP2)-4.5 future emissions scenario (Fig. 1*A*) (49). Extending out to the year 2069, ARISE-SAI includes 10 ensemble members, each initiated from slightly different initial conditions to enable quantification of the irreducible uncertainty arising from internal climate variability (e.g., 50). The 1.5 °C threshold is relevant for global policy discourse in part because this is a global mean temperature increase that is considered both an important Earth system threshold as well as a key focus of global climate policy negotiations enshrined in the United Nations’ Paris Agreement (51). The fact that ARISE-SAI simulates SAI deployment that stabilizes global temperature at 1.5 °C while also representing the effect of internal variability via a substantial number of ensemble members makes ARISE-SAI a useful testbed for probing the possibility of perceived failure of climate intervention.

## Results

Increases in greenhouse gas concentrations and other anthropogenic forcings under the SSP2-4.5 scenario drove increases in temperatures globally (Fig. 1*A*), as seen in the forced (ensemble-mean) response during the 2015 to 2034 predeployment period of ARISE-SAI (Fig. 1*B*). Visualizing the ensemble mean reduced many of the effects of internal climate variability, even though an ensemble of more than 10 members is likely needed to fully remove such effects regionally (e.g., 47, 52). Over the longer postdeployment period of 2035 to 2069, the ensemble mean exhibited a clear picture of temperatures generally holding steady throughout the rest of the simulation (Fig. 1*A*), indicative of SAI acting to stabilize temperatures even regionally (Fig. 1*C*). In reality, however, any area’s actual climate trajectory will be a combination of both the forced response and internal climate variability, which would be analogous to a single ensemble member (Fig. 1 *D* and *E*) rather than the ensemble mean.

Focusing on the decade prior to SAI deployment (“predeployment decade”; 2025 to 2034), any ensemble member (e.g., member #9) will exhibit a large range of temperature trends regionally under SSP2-4.5 (Fig. 1*D*), even though the forced response is overwhelmingly warming. This is because internal climate variability can drive short-term trends in temperature that can partially mask (or augment) the longer-term, forced trend. What is perhaps less appreciated is that internal climate variability can similarly mask the effects of SAI on a regional scale. In the decade following continuous SAI deployment (“postdeployment decade”; 2035 to 2044), ensemble member #9 exhibited warming temperatures over 49% of the land surface (Fig. 1*E*), where warming is defined as decadal temperature trends larger than 0.1 °C/decade. This trend threshold was chosen to reflect the approximate warming over the observational record (53); temperature trends less than this are referred to here as “not warming,” since they capture both cooling as well as small positive trends. Thus, the effects of internal climate variability can cause the magnitude of regional warming trends in the postdeployment decade to far exceed the forced trend from SAI.

**Fig. 2.**
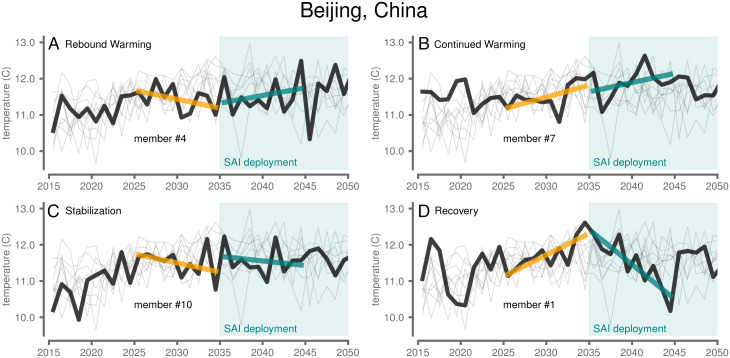
Predeployment and postdeployment surface temperature trends for Beijing, China. (*A–D*) Each panel highlights a different ensemble member denoted in each panel by the thick black line, with the other nine members shown as thin gray lines. SAI deployment was initiated in the year 2035 (teal shading). Ten-year linear best-fit lines are shown for 2025 to 2034 (orange) and 2035 to 2044 (teal).

Beijing, China, provides an example of how a single region can experience each of the four archetypal responses under different individual realizations of the ARISE-SAI experiment (Fig. 2). Ensemble member #1 exhibited the Recovery archetype (Fig. 2*D*), where SAI would potentially be labeled a success in that the perception of temperature change would swing from an increase in local temperature prior to deployment to a stabilization or decrease in temperature after deployment. However, in member #4, Beijing experienced Rebound Warming (Fig. 2*A*), with cooling over the predeployment period followed by warming over the postdeployment period. Likewise, in member #7, Beijing experienced Continued Warming (Fig. 2*B*), with substantial warming during both the pre- and postdeployment decades.

**Fig. 3.**
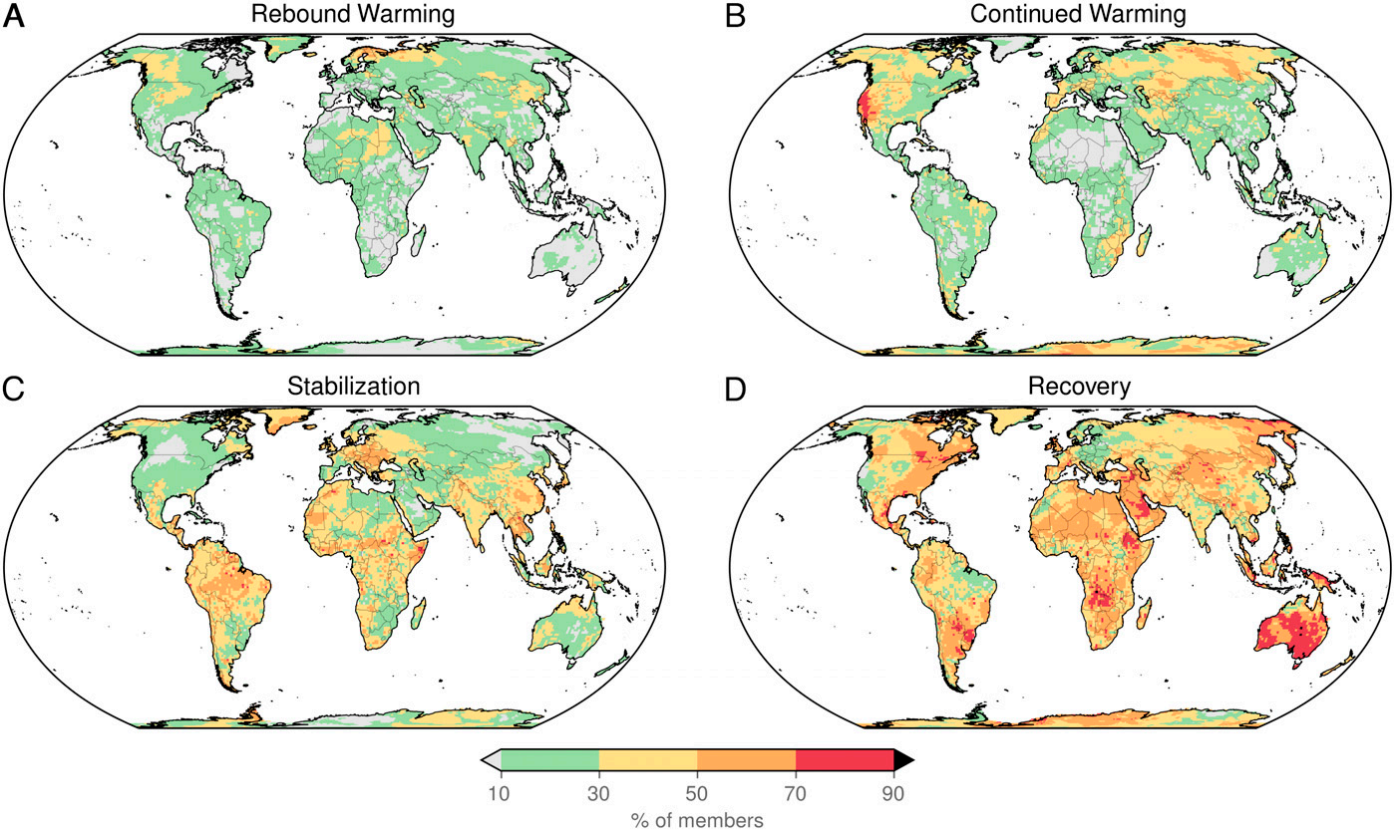
Archetypal regional responses to ARISE-SAI. (*A–D*) The percentage of ensemble members that exhibited specific archetypal responses over the 10 y pre- and postdeployment: (*A*) Rebound Warming (not warming followed by warming), (*B*) Continued Warming (warming followed by warming), (*C*) Stabilization (not warming followed by not warming), and (*D*) Recovery (warming followed by not warming).

All four archetypal regional responses can be found across the globe, with varying percentages of the ARISE-SAI ensemble (Fig. 3). While some regions, notably Australia and parts of Africa, exhibited high probability of the Recovery archetype (Fig. 3*D*), substantial parts of the land surface experienced high probability of either Rebound Warming or Continued Warming. Repeated occurrence of perceived failure in the same location across multiple ensemble members can be largely understood as internal climate variability persistently masking the effect of SAI deployment (although more than 10 ensemble members would be required to completely rule out the possibility of a weak, short-term forced response to SAI itself; Fig. 1*C*).

**Fig. 4.**
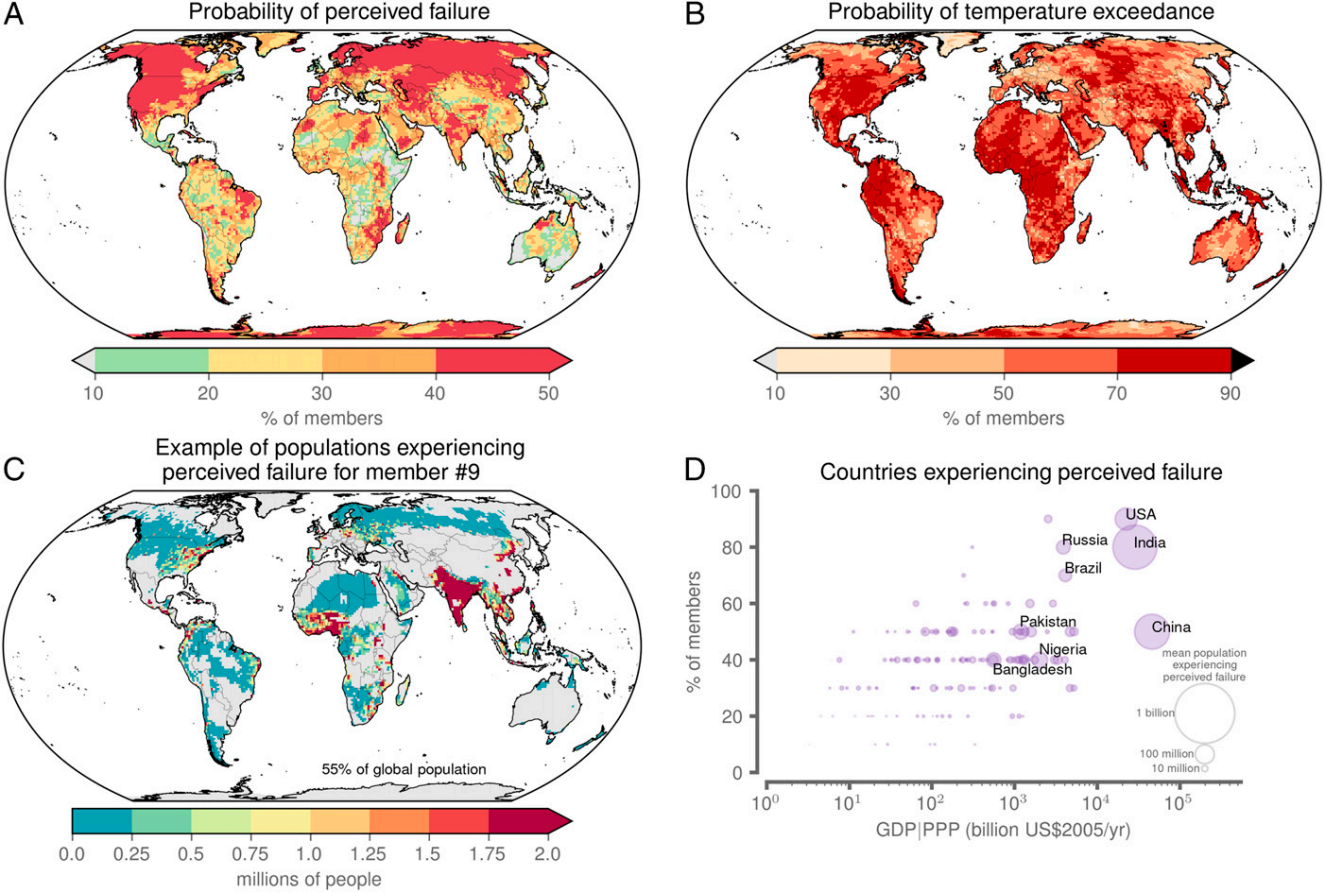
Perceived failure over the 10 y following SAI deployment under ARISE. (*A*) Probability of perceived failure over the postdeployment period, where the probability was computed as the fraction of ensemble members exhibiting warming trends. (*B*) Probability of a location exceeding its 2015 to 2034 (predeployment) maximum annual-mean temperature in the decade following SAI deployment (2035 to 2046). (*C*) Projected number of people at each location experiencing perceived failure of SAI over the postdeployment period in ensemble member #9 using projected populations for 2040. Gray denotes regions not experiencing perceived failure in that particular ensemble member. (*D*) Percentage of members with 10% or more of a country’s projected 2040 population (see *SI Appendix*, Fig. S5 for alternative population thresholds) experiencing perceived failure following SAI deployment versus the country’s projected 2040 GDP in units of PPP. Circled area corresponds to the projected 2040 population experiencing perceived failure averaged across ensemble members.

Aggregating the occurrence of Rebound Warming and Continued Warming across all ensemble members yielded the probability (computed as the percentage of the 10 ensemble members) of internal variability leading to perceived failure of SAI in the ARISE-SAI experiment (Fig. 4 *A* and *B*). While some regions of the planet experienced near-zero probability of perceived failure under ARISE-SAI deployment, there were other regions that experienced greater than 50% probability of perceived failure. East Antarctica—a region of global importance and priority with respect to the potential for substantial changes in sea level (54)—appeared particularly prone to climate variability masking the effectiveness of climate intervention. Likewise, much of northern Eurasia and the western half of North America experienced a very high probability of perceived failure in the decade following deployment. For the case of North America, Pacific Decadal Variability—which CESM is known to simulate with high fidelity (55)—could be a key factor confounding the effects of climate intervention (*SI Appendix*, Fig. S3).

We emphasize that these results are specific to ARISE-SAI deployment, which is only one of many possible SAI deployment scenarios (e.g., 56). Regardless, they suggest that internal variability in the climate system, whether arising from random noise in the atmosphere or oceans (57) or from potentially predictable coupled ocean-atmosphere modes of variability, can effectively mask SAI deployment.

Our perceived failure metric relies on quantifying decadal temperature trends. However, given the myriad impacts of extreme heat on natural and human systems (27, 58), an alternative metric for the perceived effectiveness of SAI could instead be a measure of the experience of temperature extremes following deployment. We found that although the forced response in ARISE-SAI resulted in a stabilization of global temperatures (Fig. 1 *A* and *C*), it is still very likely that record hot temperatures will occur following deployment (Fig. 4*B*). For example, for broad areas of Africa, Eurasia, North America, South America, and Antarctica, at least 1 y in the decade after SAI deployment was hotter than the hottest year that occurred in 2015 to 2034. Moreover, the regions experiencing persistently high perceived failure of SAI (Fig. 4*A*) did not directly correspond to the regions experiencing extremely high mean annual temperatures (Fig. 4*B*). This finding underlines that multiple climate metrics are necessary when considering the perceived effectiveness of SAI.

Given the importance of local experiences for informing perceptions of climate change (40), we next explored the populations exposed to perceived failure of SAI in the specific ARISE-SAI deployment scenario examined here. Using gridded population data projected for 2040 in SSP2 (59, 60), we found that between 10% and 55% of the global population experienced perceived failure across the 10-member ARISE-SAI ensemble (*SI Appendix*, Fig. S4). The most severe example is shown in Fig. 4*C* for ensemble member #9, where substantial populations in India, Southeast Asia, the eastern United States, and West Africa were exposed to the potential of perceived failure over the decade following ARISE-SAI deployment.

Perceptions of climate change–related phenomena can be related to both individual local experiences as well as collective sociocultural experiences (40, 61, 62). Thus, to further explore the socioeconomic reality of perceived failure of SAI at the national level, we compared the probability of country-level perceived failure against country-level gross domestic product (GDP) in 2040 (in units of purchasing power parity; PPP) (63). All of the largest economies in the world experienced substantial probability of perceived failure in the postdeployment decade of ARISE-SAI (Fig. 4*D*). The implication is that the countries with the most geopolitical and global economic power—and perhaps those with the most financial capacity to deploy continuous SAI to manage global temperatures (64)—experienced at least a 50% probability of large populations being exposed to the potential of perceived failure of SAI. These countries also cover substantial land areas, potentially increasing the odds that internal climatic variability could mask the benefits of SAI. Yet, the fact remains that the countries that are apparently most prone to high potential of perceived failure are those with the largest populations and the largest economies.

## Discussion

The “fast” dimension of climate intervention is a notable advantage of SAI relative to other climate intervention approaches (14, 24). However, we found that substantial areas of the world could experience warming trends and extremely hot years, even after 10 y of continuous deployment in the ARISE-SAI scenario—raising the possibility that SAI may not be perceived locally as effective. Given the potential social, political, and economic costs associated with climate intervention and increasing stakes associated with a warming planet, this gap in time between deployment and local perceived effectiveness could serve to undermine the fast dimension of SAI intervention. Moreover, SAI is a technology that could potentially be deployed quickly by a small group of actors (or a single actor), owing to its relatively low cost and ease of deployment from a single location on the planet (e.g., within the borders of a single country) (35, 64).

In light of our findings, several priorities emerge for a forward-thinking SAI research agenda. First, the prevalence of perceived failure suggests countries should expect public doubt in the short-term effectiveness of SAI. The expectation of precise manipulation would be markedly inaccurate (65). Moreover, different types of SAI deployment scenarios could lead to different levels of masking (both more and less) of internal climate variability. However, this issue will also emerge in the midst of more general mitigation efforts (66), as internal climate variability will likely produce continued warming in some regions in the years following aggressive policies aimed at reducing greenhouse gas emissions—potentially leading to similar perceptions of failure in the climate policy itself (67). Thus, whether or not SAI is pursued, countries must recognize that internal climate variability will need to be anticipated and well-articulated if continued public support is desired. Furthermore, this articulation must occur amid a communication environment that is already fraught with climate-related misinformation (68).

To further explore the relevance of the perceived failure archetypes, we performed a similar analysis using data from the Geoengineering Large-Ensemble SAI experiment (69). The results provide complementary insights into SAI deployed under a much higher emissions scenario (Representative Concentration Pathway 8.5) and different stabilization targets and deployment year (deployment in the year 2020 with the main aim to keep global temperatures around 1 °C above preindustrial values). Because of this, GLENS-SAI represents a much more aggressive SAI scenario than ARISE-SAI. The GLENS-SAI results (see *SI Appendix*) again illustrate the regional significance of internal climate variability and thus further indicate that the potential for perceived failure will exist across many different SAI deployment strategies.

Given that specific regions of the planet are predisposed to the effects of large internal climate variability, such as that produced by the El Niño Southern Oscillation or the Pacific Decadal Oscillation (70), it is likely that these regions will also experience persistent masking of SAI effectiveness. Such understanding of regionally persistent masking of SAI effectiveness will complement and contribute to the growing literature on detection and attribution of deployment of climate intervention (25, 26). Further, because the possibility of perceived failure extends beyond SAI, knowledge of specific regionally persistent internal variability will benefit other climate mitigation policies, especially those contingent on public support (71).

## Conclusions

Our results highlight the need for continued research and understanding of how climate variability may mask climate intervention in the years immediately following deployment. If climate intervention is ever pursued, it will likely be for a specific social or geophysical aim. Internal climate variability, however, may mask the short-term perceived effectiveness of that intervention, including in the targeted geographical areas, ecosystems, or economic sectors for which the intervention was deployed in the first place. Our results thus suggest that the scientific community must better frame what the success of SAI—and climate intervention more broadly—looks like in the context of internal climate variability. Specifically, it will be important to understand how key global drivers of variability, such as coupled ocean-atmosphere modes operating on decadal timescales, may mask the intended results of climate intervention strategies and to what extent this masking will be predictable or detectable. Our analysis provides a foundation for that understanding and motivation for improving the ability of global policy and scientific organizations to better frame the stakes associated with the deployment of climate intervention in the future.

## Methods

### ARISE Data.

Gridded, monthly near–surface air temperature fields (variable name TREFHT) were obtained from the ensemble of simulations performed for the ARISE-SAI (49). The ARISE ensemble was simulated with the CESM, version 2 (72) using WACCM6 (Whole Atmosphere Community Climate Model Version 6) (73). We averaged together the gridded, monthly fields to produce annual-mean fields, with each field having a grid resolution of 0.94240838 degrees latitude by 1.25 degrees longitude.

The ARISE dataset includes two sets of simulations composed of 10 ensemble members each. The first set follows the SSP2-4.5 emissions scenario, while the second is identical to the first but with the inclusion of SAI beginning in the year 2035. The location and amount of aerosols released into the stratosphere each year is determined by a controller algorithm that works to keep global mean temperature, the north-south temperature gradient, and the equator-to-pole temperature gradient at values based on the 2020 to 2039 mean of the SSP2-4.5 simulations with CESM2 (WACCM6) (73). Further details about the ARISE-SAI configuration and aerosol injection strategy are provided in ref. 49.

### Probability of Perceived Failure.

Decadal trends of annual mean temperature at each grid point were computed using linear, least-squares regression over two 10-y periods: 1) the predeployment decade (2025 to 2034) and 2) the postdeployment decade (2035 to 2044). Since SAI under ARISE is designed to stabilize global-mean temperature (not to reverse the warming trend and induce cooling), we defined “warming” as any decadal trend that exceeded 0.1 °C per decade. A warming threshold of 0.1 °C per decade was chosen to reflect the approximate warming we have thus far experienced over the observational record (53). All trend magnitudes less than this were considered “not warming.” We thus classified each of the ensemble members, for each location, as falling into one of the four archetypes of perceived success of climate intervention based on the pre- and/or postdeployment trends: 1) Rebound Warming (i.e., no warming followed by warming); 2) Continued Warming (i.e., warming followed by more warming); 3) Stabilization (i.e., no warming either before or after deployment); and 4) Recovery (i.e., warming followed by no warming). The combination of Rebound Warming and Continued Warming represented the experience of potential perceived failure, as both exhibited warming trends over the postdeployment decade that exceeded 0.1 °C per decade. The probability of perceived failure was then computed as the percentage of ensemble members (out of 10) that experienced perceived failure at each location.

### Populations and Country-Level Statistics for Those Experiencing Perceived Failure.

Projected, gridded population data for the year 2040 were downloaded from the Socioeconomic Data and Applications Center (SEDAC) for SSP2 (https://sedac.ciesin.columbia.edu/data/collection/popdynamics/maps/services). The SEDAC data were downloaded in netcdf format at a resolution of one-eighth of a degree and then regridded to the ARISE/CESM2 grid using the sum function. The global population was perfectly conserved in this regridding process. The population experiencing perceived failure was then computed as the sum of the populations at each grid point where the postdeployment decade exhibited warming trends greater than 0.1 °C. Projected GDP (in units of PPP) data for the year 2040 under SSP2 were downloaded as shapefiles from the International Institute for Applied Systems Analysis at the country level (https://tntcat.iiasa.ac.at/SspDb/dsd?Action=htmlpage&page=10). Temperature trends, projected population, and projected GDP were then calculated within each country boundary using the python packages *regionmask* and *geopandas.*

Fig. 4*D* includes the percentage of members with 10% or more of a country’s projected 2040 population experiencing perceived failure following SAI deployment. *SI Appendix*, Fig. S5 displays results for the same analysis using alternative population thresholds (i.e., 5%, 10%, 25%, and 50%).

### Probability of Exceeding Predeployment Maximum Temperature.

For each grid point, we computed the maximum annual-mean temperature across all available years prior to SAI deployment (2015 to 2034). This was done for each ensemble member separately to simulate perceptions within each individual realization of the climate system. The probability of exceeding the predeployment maximum temperature was then defined as the number of ensemble members (out of 10) that exceeded their predeployment maximum in the decade following deployment (2035 to 2044).

## Supplementary Material

Supplementary File

## Data Availability

All study data are included in the article and/or *SI Appendix*. The manuscript will be submitted in parallel to the EarthArXiv preprint server, under a CC BY 4.0 license. All ARISE and GLENS data are publicly available (see information for access): https://www.cesm.ucar.edu/projects/community-projects/ARISE-SAI/ and http://www.cesm.ucar.edu/projects/community-projects/GLENS/. Population and GDP data can be downloaded at https://sedac.ciesin.columbia.edu/data/collection/popdynamics/maps/services and https://tntcat.iiasa.ac.at/SspDb/dsd?Action=htmlpage&page=10. Code is available on GitHub at https://github.com/eabarnes1010/arise_perceived_failure (74) and is archived on Zenodo at the following DOI: https://doi.org/10.5281/zenodo.7072436 (75).
