# Supplementary material for: Potential for perceived failure of stratospheric aerosol injection deployment
Source: Proc Natl Acad Sci U S A. 2022 Sep 27;119(40):e2210036119. doi: 10.1073/pnas.2210036119 (PMC9546631; doi:10.1073/pnas.2210036119)
Supplement: Supplementary File [file pnas.2210036119.sapp.pdf]

PNAS

Supplementary Information for

Potential for Perceived Failure of Stratospheric Aerosol Injection Deployment

Patrick W. Keys[1,2*], Elizabeth A. Barnes[1], Noah S. Diffenbaugh[3], James W. Hurrell[1] and Curtis M. Bell [4]

[1] Department of Atmospheric Science, Colorado State University
[2] School of Global Environmental Sustainability, Colorado State University
[3] Doerr School of Sustainability, Stanford University
[4] International Programs Department, United States Naval War College

***Corresponding Author:** Patrick W. Keys

Email: patrick.keys@colostate.edu

This PDF file includes:

Figures S1 to S5
Supplemental Methods
Figures S6 to S7

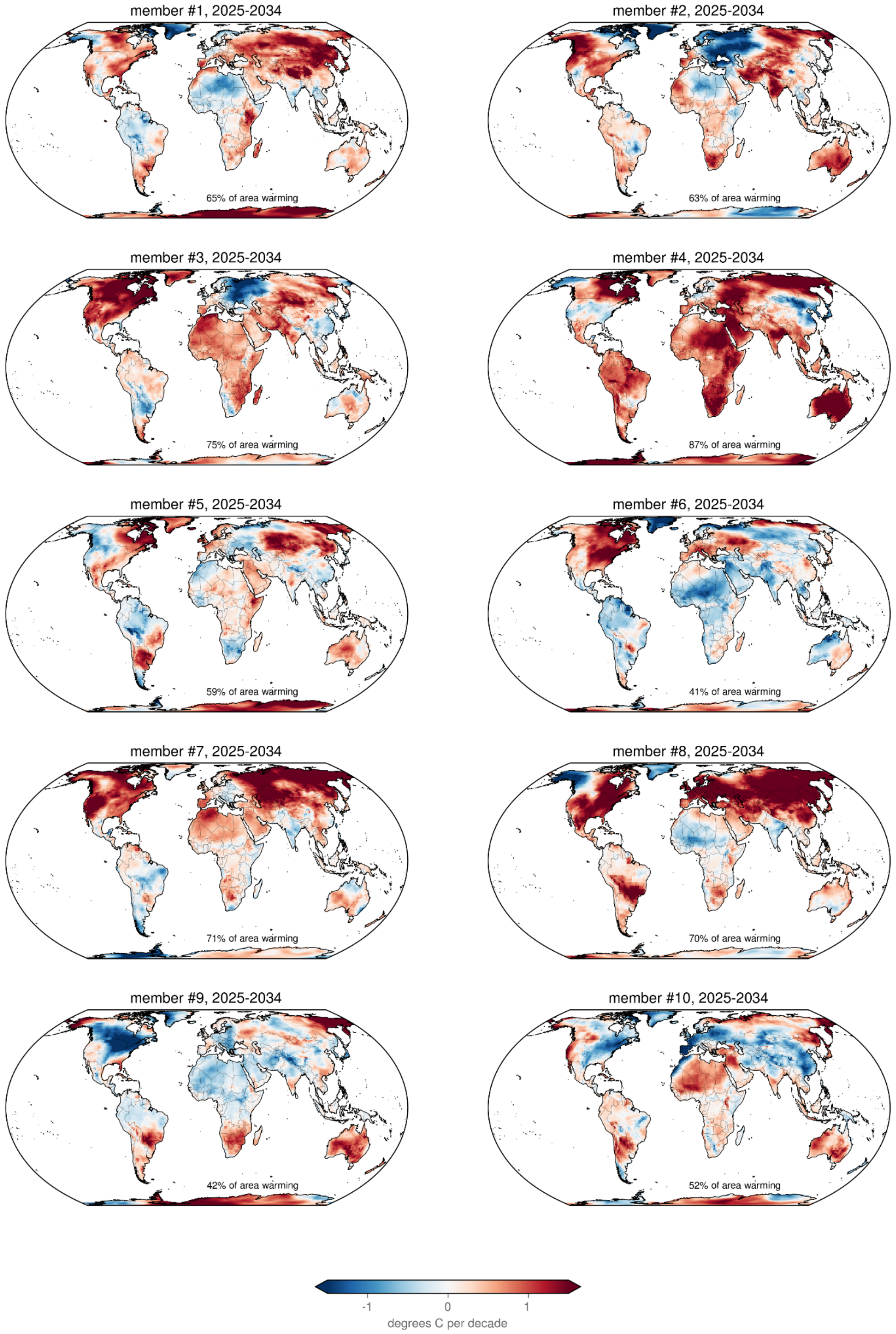

Fig. S1. As in Figure 1D but for all 10 ensemble members.

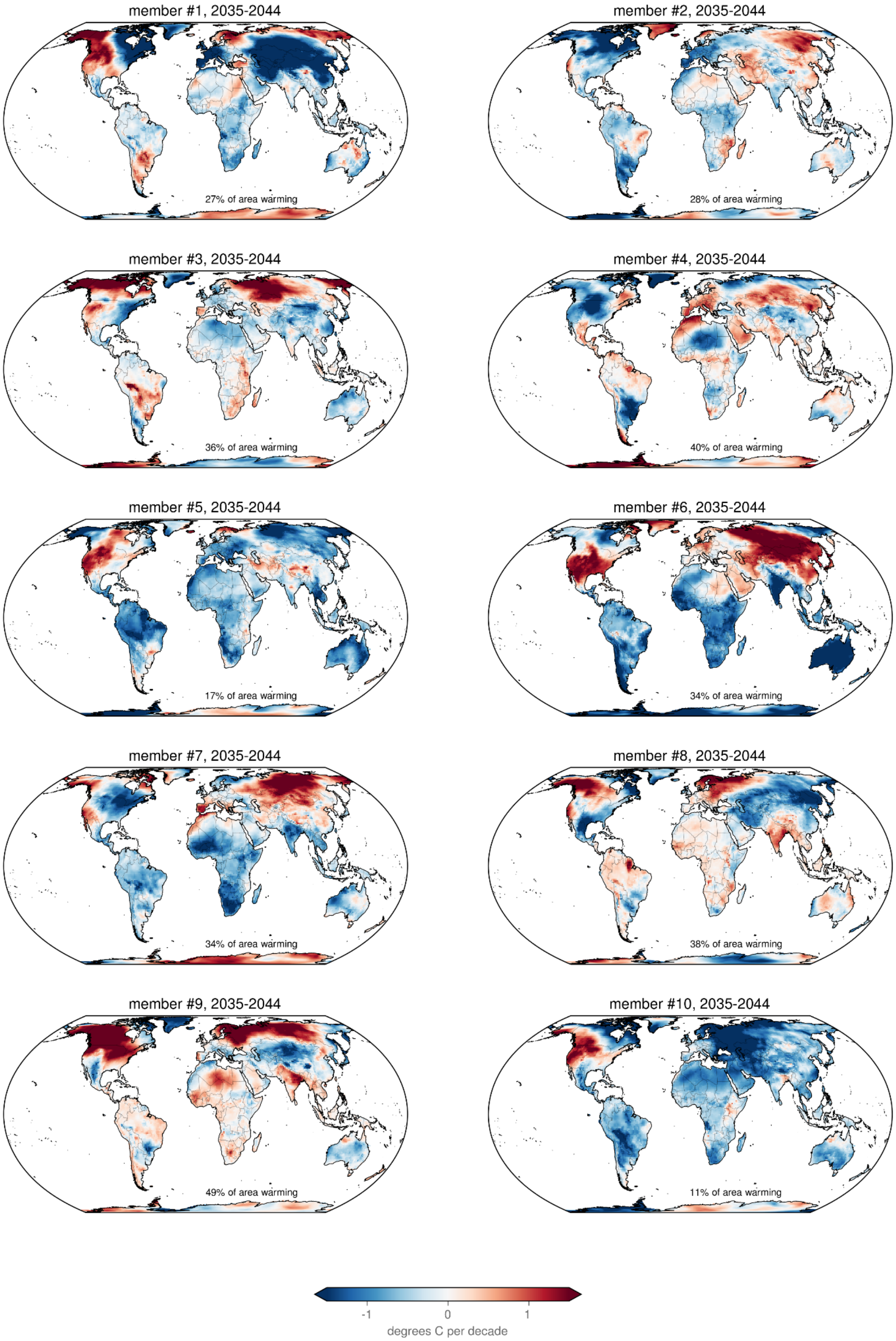

Fig. S2. As in Figure 1E but for all 10 ensemble members.

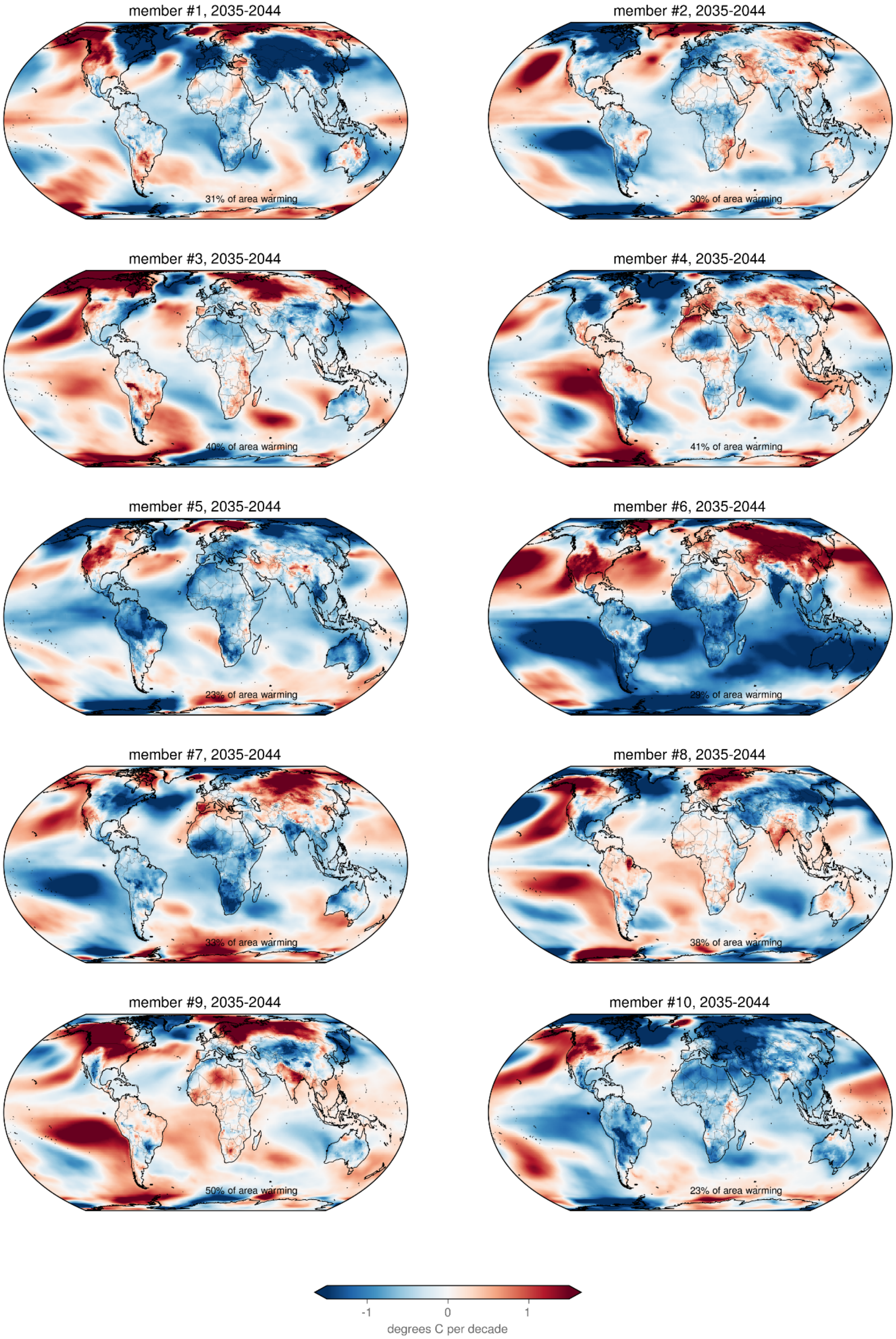

Fig. S3. As in Fig. S2 but including temperatures over the oceans.

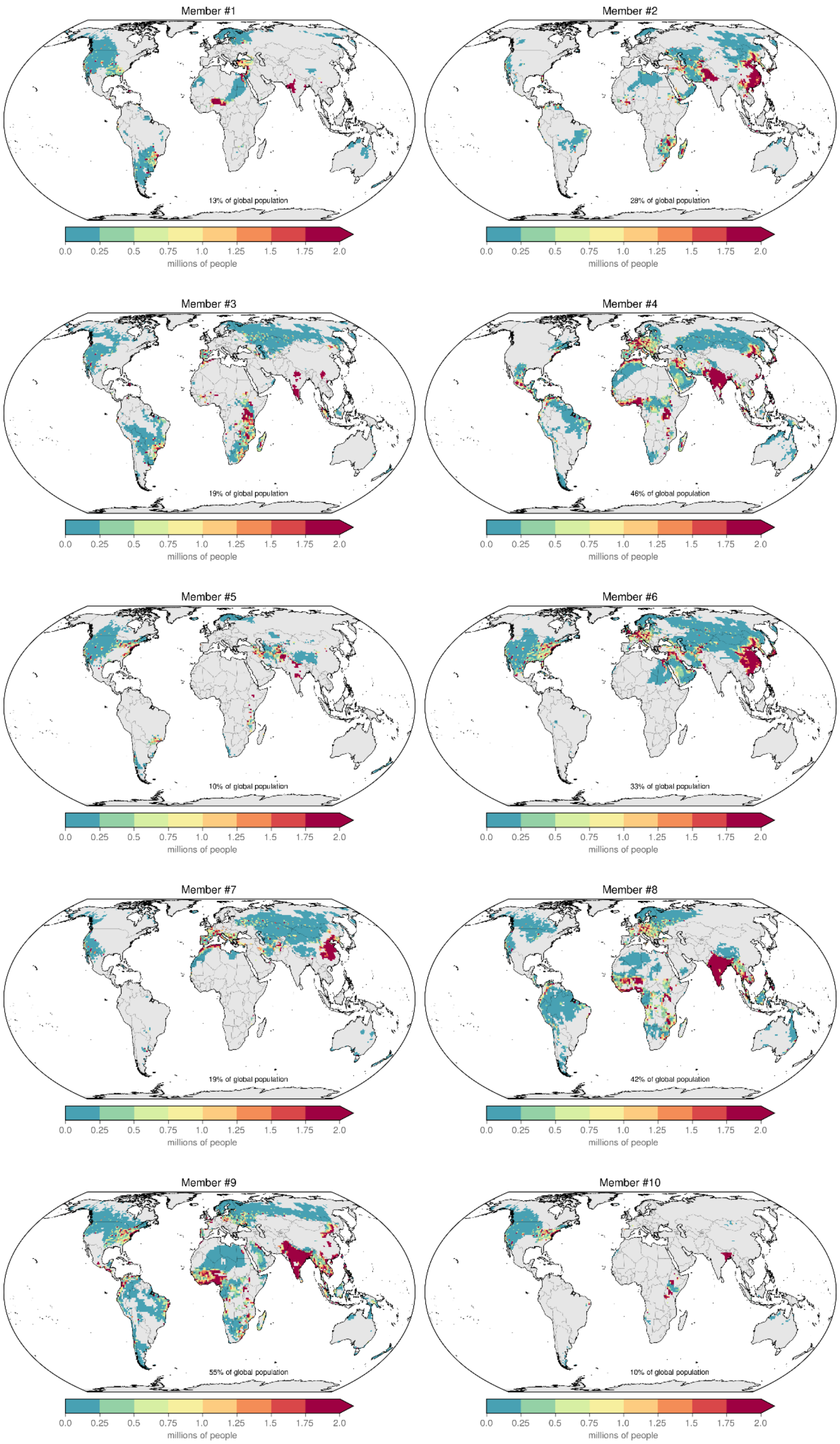

Fig. S4. As in Fig. 4A but for all 10 ensemble members.

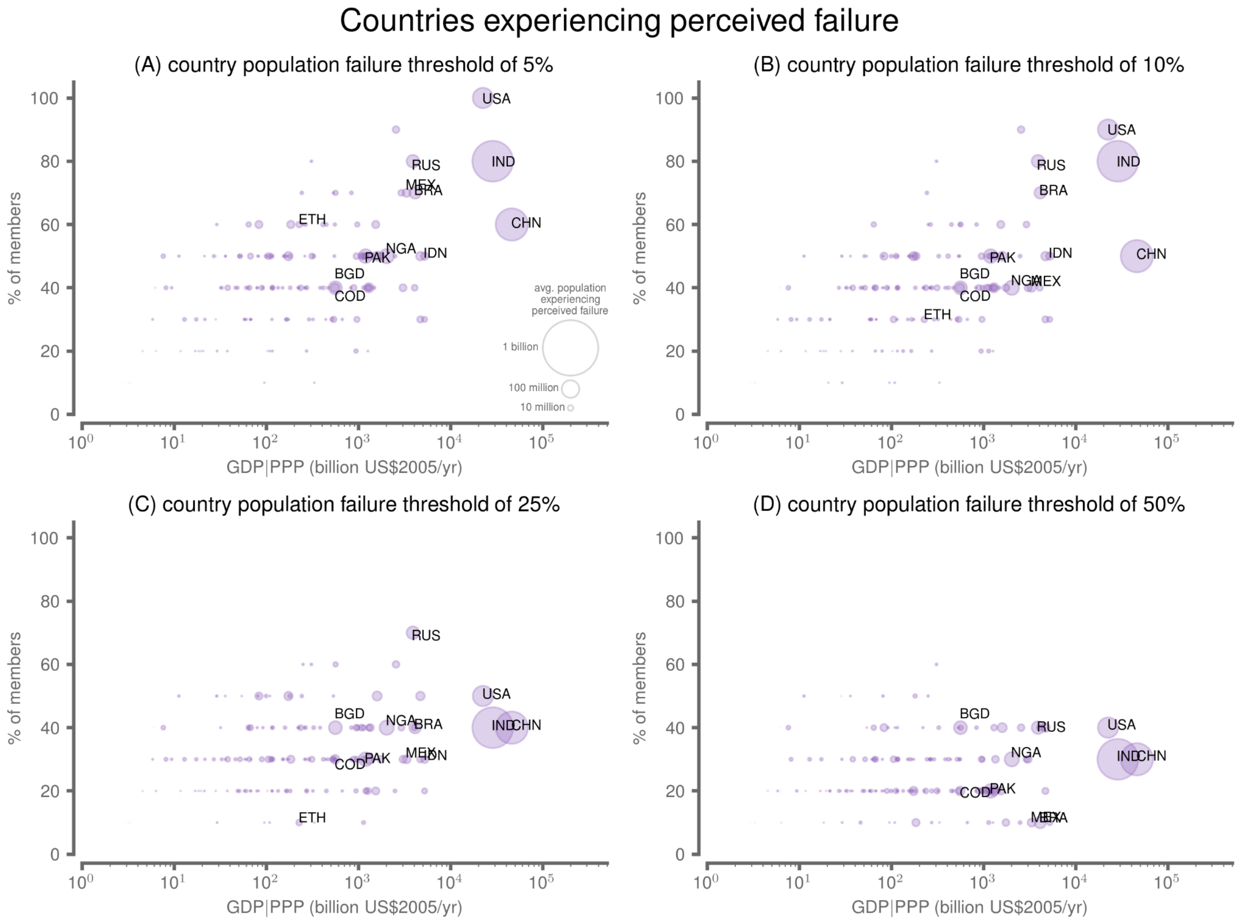

Fig. S5. As in Fig. 4B but for different population failure thresholds. The 10% threshold shown here in panel **(B)** is what is displayed in the main text.

Supplemental Methods

GLENS Data

Gridded, monthly near surface air temperature fields (variable name TREFHT) were obtained from the ensemble of simulations performed for the Stratospheric Aerosol Geoengineering Large Ensemble (GLENS-SAI) project (73). The GLENS-SAI ensemble was simulated with the Community Earth System Model, version 1, as described in (57). We average together the gridded, monthly fields to produce annual-mean fields, with each field having a grid resolution of 0.9 degrees latitude by 1.25 degrees longitude.

The GLENS-SAI data set includes two sets of simulations composed of twenty one ensemble members each. The first set follows the RCP8.5 emissions scenario while the second is identical to the first but with the inclusion of stratospheric aerosol injection (SAI) beginning in the year 2020. The location and amount of aerosols released into the stratosphere each year is determined by a controller algorithm that works to keep global mean temperature, the north-south temperature gradient, and the equator-to-pole temperature gradient at values based 2020. The 2020 mean conditions are calculated based on the first 13 members of the RCP8.5 scenario simulations. Further details about the GLENS-SAI configuration and aerosol injection strategy are provided in (73).

Probability of perceived failure

Decadal trends of annual mean temperature at each gridpoint are computed using linear, least-squares regression over two ten-year periods: (1) the pre-deployment decade (2010-2019) and (2) the post-deployment decade (2020-2029). Since SAI under GLENS is designed to stabilize global-mean temperature (not to reverse the warming trend and induce cooling), we define “warming” as any decadal trend that exceeds 0.1°C per decade. A warming threshold of 0.1°C per decade is chosen to reflect the approximate warming we have thus far experienced over the observational record (NOAA National Centers for Environmental Information, published online January 2021). All trend magnitudes less than this are considered “not warming”. We thus classify each of the ensemble members, for each location, as falling into one of the four archetypes of perceived success of climate intervention, based on the pre- and/or post-deployment trends: 1) Rebound Warming (i.e. no warming followed by warming); 2) Continued Warming (i.e. warming followed by more warming); 3) Stabilization (i.e. no warming either before or after deployment); and, 4) Recovery (i.e. warming followed by no warming). The combination of Rebound warming and Continued warming represent the experience of potential “perceived failure”, as both exhibit warming trends over the post-deployment decade that exceed 0.1°C per decade. The probability of perceived failure is then computed as the percent of ensemble members (out of 20) that experience perceived failure at each location.

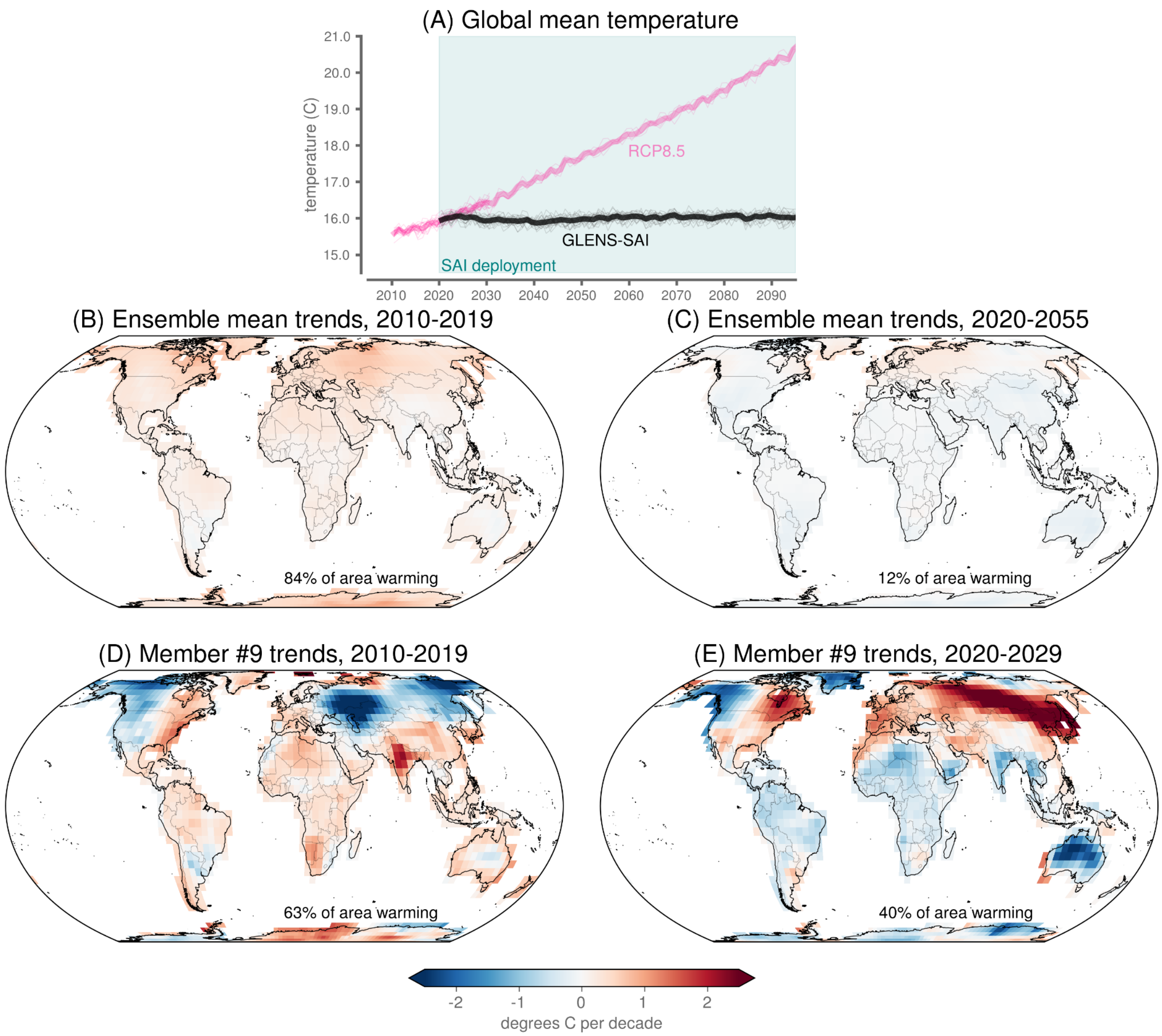

Figure S6. Surface temperature trends. (A) Global mean surface temperature. Gray lines denote individual ensemble members and the black line denotes the ensemble mean. **(B,C)** Ensemble-mean trends over **(B)** 2010-2019 under RCP8.5 and **(C)** 2020-2055 with GLENS SAI deployment. **(D,E)** Trends over the **(D)** pre-deployment decade and **(E)** post-deployment decade for ensemble member #9. **(B-D)** The percentage in the bottom of the maps denotes the percentage of land area that exhibits warming trends as defined in the text. Similar figure as in Figure 1 of the Main text.

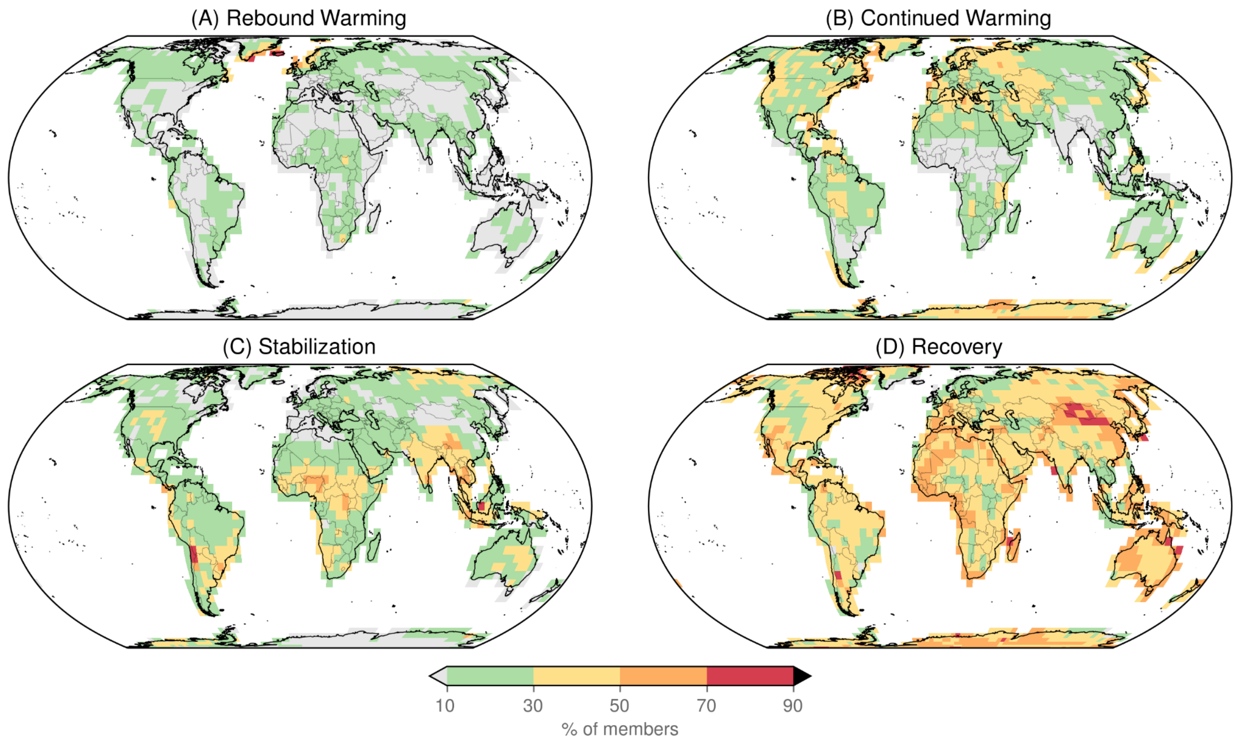

Figure S7. Archetypal regional responses to GLENS-SAI. The percent of ensemble members that exhibit specific archetypal responses over the ten years pre- and post-deployment: **(A)** Rebound Warming (not warming followed by warming), **(B)** Continued Warming (warming followed by warming), **(C)** Stabilization (not warming followed by not warming) and **(D)** Recovery (warming followed by not warming). Similar figure as in Figure 3 in the Main text.

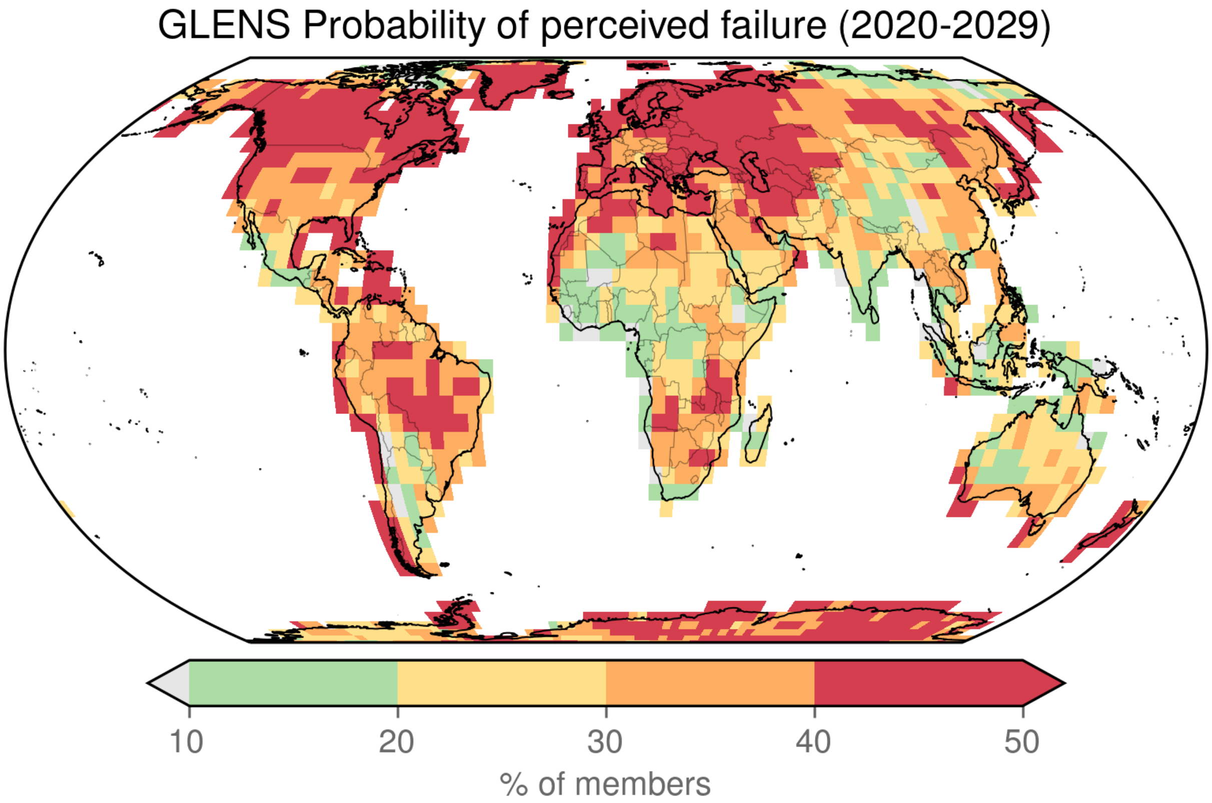

Figure S7. Probability of perceived failure under GLENS-SAI over the post-deployment period, where the probability is computed as the fraction of ensemble members exhibiting warming trends (similar to Figure 4A in the Main text).
